# Branch retinal vein occlusion associated with quetiapine fumarate

**DOI:** 10.1186/1471-2415-11-24

**Published:** 2011-08-25

**Authors:** Ku Chui Yong, Tan Aik Kah, Yeap Thye Ghee, Lim Chee Siang, Mae-Lynn Catherine Bastion

**Affiliations:** 1 Department of Ophthalmology, Faculty of Medicine, Universiti Kebangsaan Malaysia Medical Centre (UKMMC), Jalan Yaacob Latif, Bandar Tun Razak, Cheras, 56000 Kuala Lumpur, Malaysia; 2 Department of Ophthalmology, Faculty of Medicine and Health Sciences, Universiti Malaysia Sarawak (UNIMAS), Lot 77, Seksyen 22, Kuching Town Land District, Jalan Tun Ahmad Zaidi Adruce, 93150 Kuching, Sarawak, Malaysia; 3 Emergency Department, Faculty of Medicine, Universiti Kebangsaan Malaysia Medical Centre (UKMMC), Jalan Yaacob Latif, Bandar Tun Razak, Cheras, 56000 Kuala Lumpur, Malaysia

## Abstract

**Background:**

To report a case of branch retinal vein occlusion in a young adult with bipolar mood disorder treated with quetiapine fumarate.

**Case Presentation:**

A 29 years old gentleman who was taking quetiapine fumarate for 3 years for bipolar mood disorder, presented with sudden vision loss. He was found to have a superior temporal branch retinal vein occlusion associated with hypercholesterolemia.

**Conclusion:**

Atypical antipsychotic drugs have metabolic side effects which require regular monitoring and prompt treatment.

## Background

Retinal vein occlusions (RVOs) frequently occur in the elderly in association with atherosclerosis. Hypertension is the commonest cause of RVOs in such population. In young adults, RVOs are associated with vasculitis and coagulopathies. Extensive work-up is required as both vasculitis and coagulopathies can lead to severe systemic morbidity and mortality. We describe a case of branch retinal vein occlusion in a young adult who has been taking quetiapine fumarate for 3 years.

## Case Presentation

A 29 years old gentleman was referred from the emergency department with the complaint of sudden painless vision loss of his left eye for the past 1 week. Since onset, he experienced progressive generalized blurring of the central vision. There was neither photopsia nor floaters. Systemic review was not significant. He has no symptoms and signs of systemic vasculitis such as rashes, joint pains or mucosal surface ulcers. He was diagnosed of bipolar mood disorder in 2008, and was treated with oral quetiapine fumarate 100 mg daily. His bipolar mood disorder responded well to quetiapine fumarate without any side effects. Sexual history was not significant and he has no history of substance abuse or smoking. There was no family history of vascular events as well.

He was a medium built individual with a body mass index of 24.83 kg/m^2 ^(height 165.5 cm, body weight 68 kg). His body weight prior to quetiapine treatment was 62 kg. Blood pressure was 122/74 mmHg with a regular pulse rate of 80 beats per minute. The visual acuity of his left eye was 0.33, with near visual acuity of N24 at 33 cm. The right eye had visual acuity of 1.0 and near vision of N6 at 33 cm. Confrontation test revealed a left central scotoma. Relative afferent pupillary defect was absent. Anterior segment examination for both eyes was normal. The intraocular pressure was 16 mmHg bilaterally.

**Figure 1:**
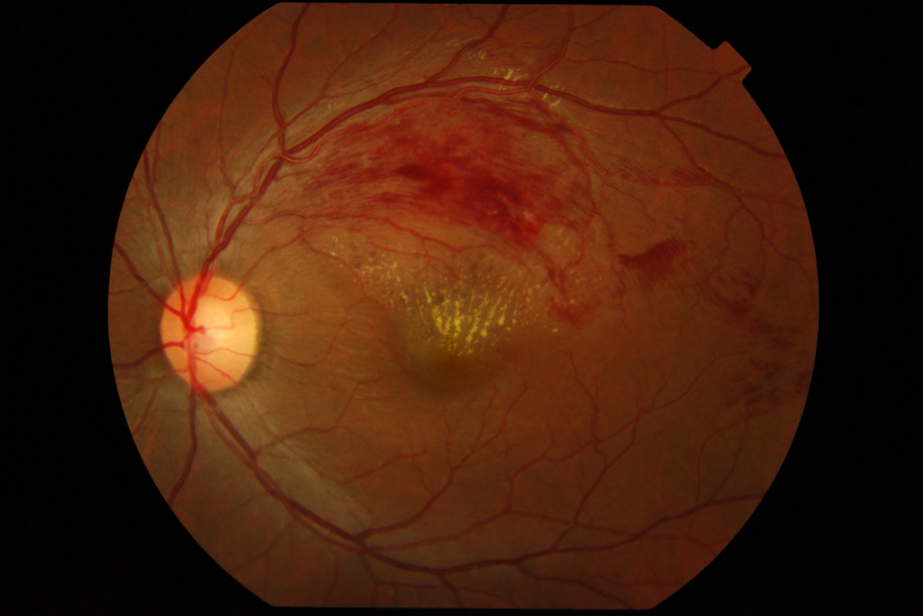
**Fundus photograph of the left eye; macula centered**.

**Figure 2:**
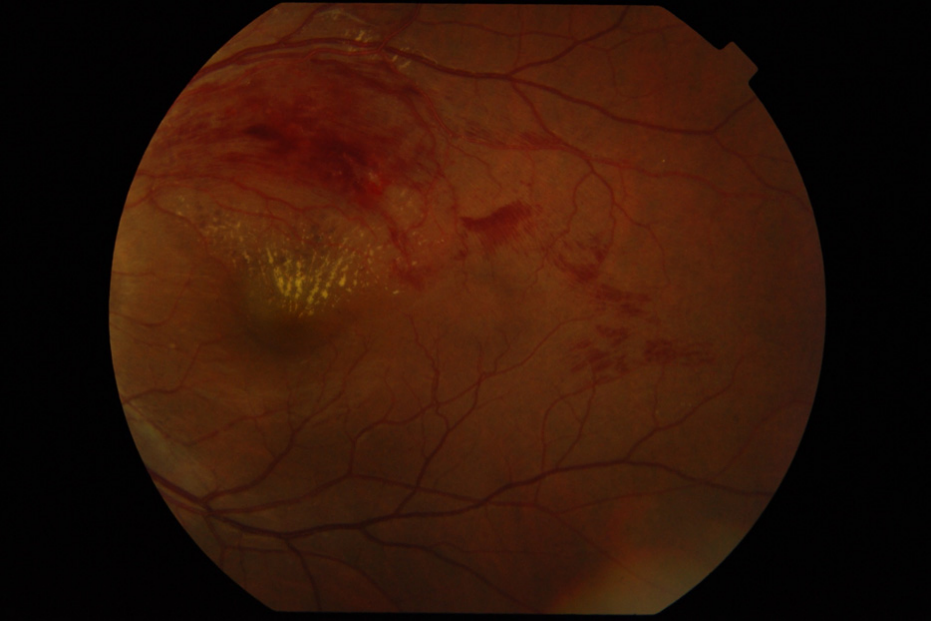
**Fundus photograph of the left eye; temporal retinal field**.

**Figure 3:**
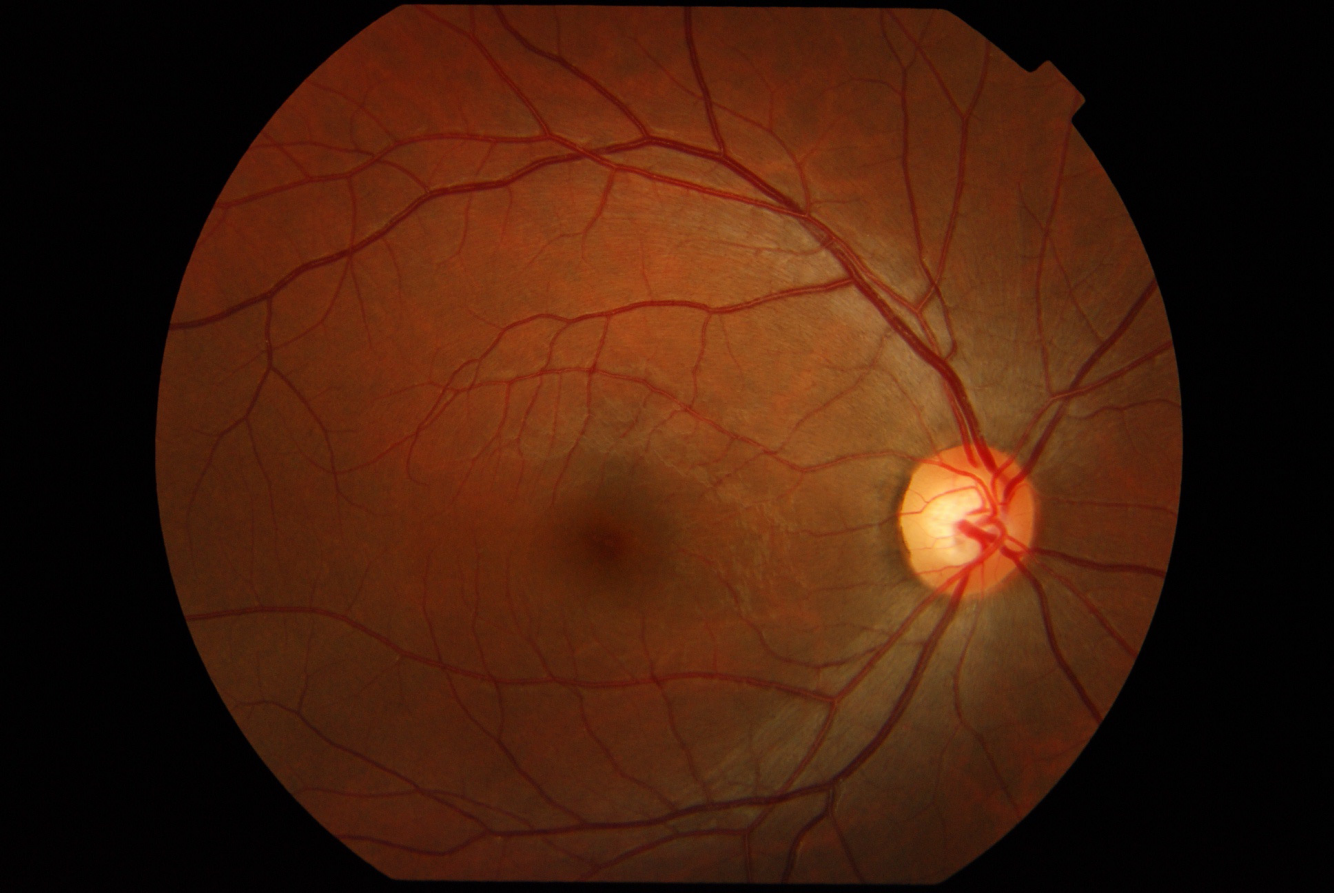
**Fundus photograph of the right eye**.

Posterior segment examination of the left eye revealed flame-shaped retinal haemorrhages along an arcuate course, corresponding to the supero-temporal retinal nerve fibre layer. Macular oedema involved the fovea and was associated with partial macular star due to hard exudates deposition. Intraretinal pigmentary spots with adjacent typical red blot hemorrhages believed to be resolving blot hemorrhages were seen. The patient has never had any laser treatment before. The supero-temporal retinal vein was dilated and tortuous along its entire course (Figure 1). The arterio-venous ratio was 2:3 infero-temporally but was 1:3 supero-temporally. There were increased arteriole reflexes with arterovenous nicking which was confined only to the supero-temporal retinal artery of the left eye. The optic disc was pink with well-defined margin and cup-disc ratio of 0.5. There was another area of flame-shaped retinal haemorrhages, two-disc diameter temporal to the fovea. The retinal haemorrhages were oriented along the distal infero-temporal retinal nerve fiber layer but did not begin at the retinal artery-vein intersection. The infero-temporal retinal vein was neither dilated nor tortuous (Figure 2). There were no cotton wool spots, the vitreous was clear, and there was no evidence of retinal periarteritis or periphlebitis. Posterior segment findings of the right eye were not significant (Figure 3).

**Figure 4:**
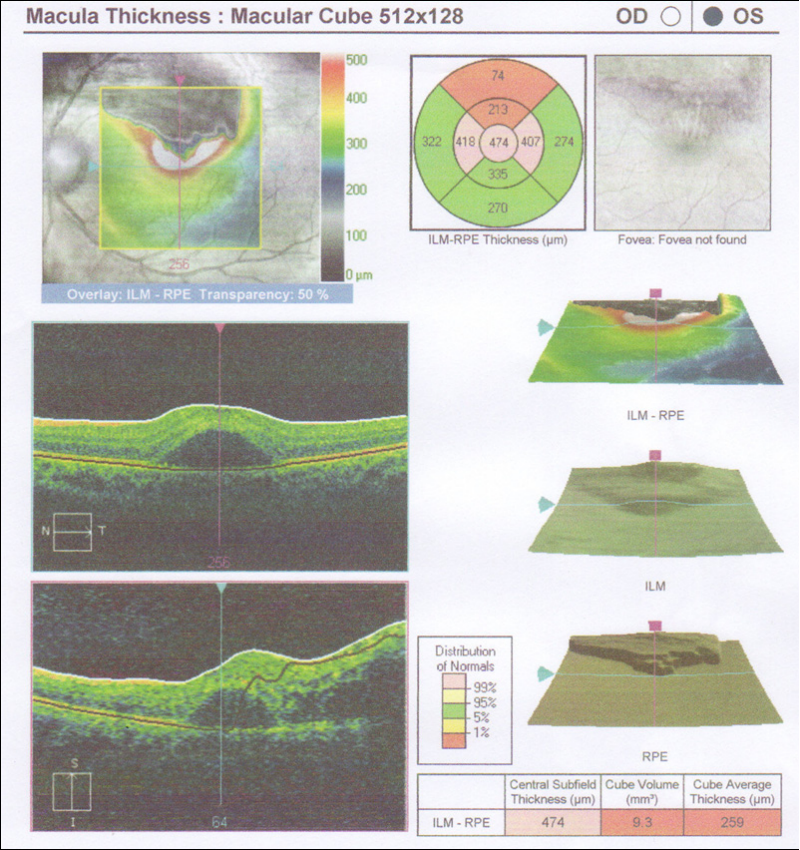
**Optical coherence tomography of the left eye**.

Optical coherence tomography (Heidelberg Spectralis ^® ^Tracking Laser Tomography) revealed the thickness of the superior half of the fovea was increased, with central serous neurosensory retinal detachment (Figure 4). Full blood count, renal profile, liver function test and thyroid function test were all normal. Erythrocyte sedimentation rate was 3 mm/hour, C-reactive protein was 0.05 mg/dl. Connective tissue screening test (antinuclear antibodies, anti double-stranded DNA antibodies, rheumatoid factor), Venereal Disease Research Laboratory test and enzyme-linked immunosorbent assay for human immunodeficiency virus were all negative. Fasting blood sugar was 4.7 mmol/L. However, annual lipid profile showed progressive derangement. The total cholesterol, triglyceride and LDL-cholesterol levels showed an increasing trend. While the HDL-choleterol levels were progressing in the opposite direction (Table 1). Chest radiography was normal.

**Table 1:** Summary of the patient's lipid profile

	January 2008	February 2010	March 2011	Normal range
Total Cholestrol (mmol/L)	4.28	4.46	5.09	<5.7

Triglycerides (mmol/L)	1.11	1.63	1.99	<1.4

HDL (mmol/L)	1.27	0.87	0.76	>1.2

LDL (mmol/L)	2.51	2.84	3.43	<3.8

The patient was diagnosed to have left major superior temporal branch retinal vein occlusion complicated by macula oedema. The cause of the RVO was attributed to dyslipidaemia secondary to quetiapine fumarate. The condition was conveyed to his psychiatrist, and he was subsequently referred to the internist for the management of his dyslipidaemia. The patient was treated with oral lovastatin 20 mg daily. His lipid profile normalized after 2 months but the final visual acuity remained 0.33 and near visual acuity N24 at 33 cm due to the presence of hard exudates at the fovea.

## Discussion

Retinal vascular diseases are ocular manifestations of underlying systemic vascular disorders. The eyes are the only place in the body which allow direct visualization of the blood vessels. Therefore fundus examination offers a valuable opportunity for the early detection of occult systemic vascular disorders. Cugati et al in his analysis of 2 population-based cohorts (Beaver Dam Eye Study and Blue Mountains Eye Study) found out that participants aged less than 70 years old with retinal vein occlusion (RVO) at baseline were associated with higher cardiovascular mortality [1].

Being a part of the entity, RVO is the second most common retinal vascular disease after diabetic retinopathy. The true prevalence of retinal vein occlusive disorder is difficult to establish as many of them are asymptomatic and only diagnosed incidentally, unless it is complicated and visual disturbances manifest. Peripheral branch RVOs are asymptomatic. Symptomatic RVOs are due to macular involvement in which patients present loss of central vision. Macula oedema is the major cause of central visual loss in RVOs [2]. Nonetheless, data from longitudinal population based study has lead the Blue Mountain Eye Study to conclude the 10-year cumulative incidence of RVO to be at 1.6%, and it was greatly associated with increasing age especially those above 70 years old [3].

Although the vast majority of RVO are found in the middle aged and elderly population, it can affect young adults too. In a retrospective review of 60 cases, Lam et al found that only 1.7% of all branch retinal vein occlusions (BRVO) occurred in individual aged 49 or younger. Systemic hypertension, hyperlipidemia and increased body mass index are important risk factors for BRVO in this group of patients [4]. However, Lam et al did not test their young subjects for hypercoagulability or collagen vascular disease, and therefore they cannot comment on the need for more extensive work-up for such patients.

Quetiapine fumarate is a second generation antipsychotics that is currently in use for the treatment of many psychiatric disorders. Unlike the older phenothiazine-type drugs, these atypical antipsychotics block both the dopamine-2 receptor and the serotonin 5-HT2A receptor; hence making them a versatile group of drug to treat a wide variety of psychiatric disorders [5]. Another advantage of these second generation antipsychotics is the lower incidence of extrapyramidal side effects and tardive dyskinesia. The safety of these new atypical antipsychotics compared to the older phenothiazines is uncertain as they can cause serious adverse drug reactions.

Atypical antipsychotic agents increase the risk of glucose intolerance, dyslipidaemia and weight gain [6]. Quetiapine fumarate has been associated with high triglycerides in 23 percent and high cholesterol in 16 percent of patients taking it [7]. Olfson et al found that treatment with quetiapine was associated with increased risk of incidence of hyperlipidaemia (odds ratio:1.52, 95% CI: 1.40-1..65) [8]. Our patient had a normal baseline laboratory results, but went on to develop dyslipidaemia following 2 years of quetiapine treatment. Even though there is a link between dyslipidaemia and BRVO, the evidence in this case is not very strong since the patient's dyslipidaemia was not severe. Nevertheless, we must be vigilant and intervene at the first sign of dyslipidaemia before the patient develops central retinal vein occlusion, stroke or any cardiovascular event. In 2004, the American Diabetes Association, the American Psychiatric Association, the American Association of Clinical Endocrinologists, and the North American Association for the Study of Obesity consensus statement recommended that fasting plasma glucose, lipid levels, and blood pressure should also be assessed 3 months after initiation of antipsychotic medications. In those with a normal lipid profile, repeat testing should be performed at 5-year intervals or more frequently if clinically indicated [9].

The subtle worsening dyslipidaemia in this patient resulted in a thrombogenic environment that led to the development of BRVO. The localized increased arteriole reflexes with arteriovenous nicking found only along the right supero-temporal retinal artery were believed to be the sequelae of BRVO rather than the cause of it. This is due to hypoxia which induced localized microglial cells proliferation along the right supero-temporal retinal artery [10].

There was a smaller zone of flame shaped haemorrhages at the inferior temporal arcade. Features characteristic for a branch retinal vein occlusion were absent in this area. The presence of a peripheral branch retinal vein occlusion at this area is very difficult to prove without fluorescein angiography. We did not perform fluorescein angiography at the time of diagnosis because we felt that the risk outweighed the benefit, since the confirmation of the second smaller inferior temporal BRVO will not alter our management. To the best of the authors' knowledge, this is the first case of BRVO associated with quetiapine. This case report emphasize the importance of regular monitoring and prompt intervention of any metabolic derangement in order to avert severe morbidity and mortality.

## Conclusions

RVOs in young adults require careful systemic evaluation for the presence of cardiovascular risk factors as well as to exclude hypercoagulabilities or collagen vascular diseases. The usage of antipsychotics medications require regular monitoring and prompt intervention for any metabolic side effects and adverse drug reactions.

## Consent

Written informed consent was obtained from the patient for publication of this case report and any accompanying images. A copy of the written consent is available for review by the Editor-in-Chief of this journal.

## Competing interests

The authors declare that they have no competing interests.

## Authors' contributions

KCY took complete history and performed physical examination. TAK performed exhaustive laboratory investigations. KCY and TAK conceived the association between quetiapine and BRVO. YTG was in-charge of referral to the psychiatrist. LCS was in-charge of referral to the physician. MLB was the consultant in-charge in the management of the patient. All authors were involved in literature review and drafted the discussion. All authors read and approved the final manuscript.

## Authors' information

KCY (MD), TAK (MD), YTG (MD), LCS (MD), MLB (FRSC), Associate Professor, Consultant Vitreoretinal Surgeon, Universiti Kebangsaan Malaysia Medical Centre (UKMMC)

## Pre-publication history

The pre-publication history for this paper can be accessed here:

http://www.biomedcentral.com/1471-2415/11/24/prepub
